# Utilization of Small Dense Low-Density Lipoprotein Cholesterol Testing in Korean Patients Visiting Local Clinics and Hospitals

**DOI:** 10.3390/nu14153246

**Published:** 2022-08-08

**Authors:** Rihwa Choi, Sang Gon Lee, Eun Hee Lee

**Affiliations:** 1 Department of Laboratory Medicine, Green Cross Laboratories, Yongin 16924, Korea; 2 Department of Laboratory Medicine and Genetics, Samsung Medical Center, Sungkyunkwan University School of Medicine, Seoul 06351, Korea; 3 Green Cross Laboratories, Yongin 16924, Korea

**Keywords:** dyslipidemia, hypercholesterolemia, low-density lipoprotein, small dense LDL, Korea

## Abstract

Small dense low-density cholesterol (sdLDL) has been the focus of studies due to its potential as an independent risk factor for atherosclerotic cardiovascular diseases. We aimed to investigate the utilization of sdLDL testing by LDL particle size analysis and the prevalence of an sdLDL predominant phenotype in Korean adult patients by visiting local clinics and hospitals. Among 9222 Korean adults (4577 men and 4645 women) with a median age of 62.8 years (interquartile range, IQR 54.5 to 71.8 years) undergoing lipid profile testing using LDL particle size analysis, the prevalence of hypercholesterolemia (total cholesterol ≥ 240 mg/dL), hypo HDL cholesterolemia (<40 mg/dL), and hyper LDL cholesterolemia (≥160 mg/dL) was 7.8%, 12.9%, and 0.5%, respectively. The overall prevalence of the sdLDL predominant non-A phenotype of LDL was 46.8% of study subjects. Approximately 32.8% of the study subjects possessed lipid test results that did not exhibit increased risk except for sdLDL (only the sdLDL predominant non-A phenotype as a risk factor). In Korea, sdLDL testing was utilized in patients whose LDL cholesterol level was not increased. Future studies to clarify the clinical significance of this test in the Korean population are needed.

## 1. Introduction

Hyperlipidemia is a global concern due to its association with atherosclerotic cardiovascular diseases [1,2,3]. Among the major lipoproteins in blood including chylomicrons, very low-density lipoprotein (VLDL), intermediate-density lipoprotein (IDL), low-density lipoprotein (LDL), and high-density lipoprotein (HDL), increased LDL is a major analytical marker for diagnosis and management of atherosclerotic cardiovascular diseases [3,4,5,6]. LDL particles have a variety of sizes and densities [4,5]. Among LDL particles, the small dense subtype (small dense LDL; sdLDL) has been the focus of studies because of its potential as a risk factor for atherosclerosis and cardiovascular events [2,4,7,8].

Metabolic pathways for lipids in the liver in concert with triglyceride availability and lipoproteins produced by stepwise modifications result in sdLDL [9,10,11]. The size of LDL particles, containing protein (apolipoprotein B-100), cholesterol, phospholipid, and triglycerides, decreases with the relative contents of free cholesteryl ester, free cholesterol, and phospholipid and increases with higher triglycerides and protein content [11,12]. The compositional alterations in LDL particles causing changes in apolipoprotein B-100 conformation on the surface of LDL lead to decreases in LDL receptor binding affinity, plasma clearance, and resistance to oxidation of sdLDL, resulting in its greater penetration ability into arterial walls [9,11,12,13]. The Justification for the Use of Statin in Prevention: An Intervention Trial Evaluating Rosuvastatin (JUPITER) study confirmed the cardiovascular heart disease risk associated with sdLDL and lipid lowering medications such as statins, fibrates, ezetimibe, niacin, proprotein convertase subtilisin/kexin type 9 (PCSK9) inhibitors, and other non-medical interventions including dietary modifications and exercise have been reported to manage the sdLDL level to reduce cardiometabolic risks [8,9,14].

The Third Report of the National Cholesterol Education Program (NCEP), Expert Panel on Detection, Evaluation, and Treatment of High Blood Cholesterol in Adults (Adult Treatment Panel III; NCEP ATP III) has introduced small LDL particles as a biomarker of atherogenic dyslipidemia, a major risk factor for metabolic syndrome [1]. Current clinical guidelines by the Japan Atherosclerosis Society (JAS) recommend considering high sdLDL level as a risk factor for atherosclerotic cardiovascular diseases [15]. The clinical guidelines for management of dyslipidemia by the Committee of Clinical Practice Guideline of the Korean Society of Lipid and Atherosclerosis (KSoLA) introduced sdLDL as a risk factor for atherosclerotic cardiovascular diseases, with increased sdLDL being a characteristic of dyslipidemia, which is associated with chronic kidney disease (CKD) [16]. Recently, a prospective Framingham Offspring study reported that sdLDL is the most atherogenic lipo-protein parameter [12], while another study performed in the United States including National Health and Nutrition Examination survey (NHANES) and Multi-Ethnic Study of Atherosclerosis (MESA) participants reported that sdLDL exhibited the strongest association with atherosclerotic cardiovascular diseases among other lipid parameters, making it helpful for improving risk stratification of patients [2].

Meanwhile, in Korea, sdLDL testing has been reimbursed by the Health Insurance Review & Assessment Service (HIRA), using an electrophoresis method, the Lipoprint LDL system, since 1 December 2016. However, limited data are available for the prevalence of patients with increased sdLDL, with limited detection resulting in an increased risk of atherosclerotic cardiovascular diseases. Since Green Cross Laboratories is a referral laboratory providing analysis for sdLDL using the Lipoprint system for samples from local clinics and hospitals throughout Korea, we aimed to investigate the prevalence of patients who exhibited increased sdLDL in a large Korean adult population. These data provide basic statistics of the population prevalence of hyperlipidemia, which can be used for public health strategies and translational research in the assessment of dyslipidemia in the Korean population.

## 2. Materials and Methods

We retrospectively investigated lipid profile results measured by the Lipoprint LDL system (Quantimetrix, Redondo Beach, CA, USA) through the laboratory information system of Green Cross Laboratories, Korea, between January 2021 and December 2021. Data from patients with missing age or sex data were excluded. Since the aim of this study was to investigate the prevalence of increased sdLDL, repeat data from the same individual were excluded. Since the test results of LDL subfraction are valid when the serum total cholesterol level is >100 mg/dL to avoid overestimation of VLDL cholesterol, data with total cholesterol ≤ 100 mg/dL were excluded. All data were anonymized before statistical analysis.

Lipids were measured using the Lipoprint LDL system according to the manufacturer’s instructions. The Lipoprint LDL system uses electrophoresis to measure the particle size of lipids, quantify subfractions from VLDL to HDL (VLDL, IDL, LDL, and HDL), and separate LDL into seven subfractions from LDL1 to LDL7 by size [16]. The cut-off value for two distinct LDL subfraction phenotypes—the large buoyant dominant phenotype (phenotype A) and sdLDL dominant phenotype (phenotype non-A)—provided by the manufacturer was a mean LDL particle size ≥ 268 Å (phenotype A).

Lipid tests were categorized according to NCEP ATP III criteria [1]. For total cholesterol, a desirable concentration < 200 mg/dL, borderline high 200–239 mg/dL, and high ≥ 240 mg/dL were applied. For HDL, low concentration < 40 mg/dL and high concentration ≥ 60 mg/dL were applied. For LDL, an optimal LDL concentration < 100 mg/dL, near optimal/above optimal 100–129 mg/dL, borderline high 130–159 mg/dL, high 160–189 mg/dL, and very high ≥ 190 mg/dL were applied.

The non-parametric method was used when appropriate to compare lipid test results by sex and age (Mann–Whitney U test). Numbers and percentages of patients with hyperlipidemia based on NCEP ATP III criteria are presented. Chi-square tests were used to compare the prevalence of hyperlipidemia categorized by NCEP ATP III criteria and of LDL subfraction phenotype by sex and age group. A value of *p* < 0.05 was considered statistically significant with the MedCalc statistical software version 20.110 (MedCalc Software bv, Ostend, Belgium). A Venn diagram for subjects having major risk factors for coronary heart disease (total cholesterol ≥ 240 mg/dL, LDL ≥ 160 mg/dL, HDL < 40 mg/dL, and phenotype non-A predominant sdLDL with a mean LDL particle size < 268 Å) was created using Venny v.2.1.0 (Oliveros, J.C. 2007–2015), an interactive tool for comparing lists with Venn diagrams (https://bioinfogp.cnb.csic.es/tools/venny/index.html, accessed on 18 July 2022). Ethical approval for this study was obtained from the Institutional Review Board of Green Cross Laboratories (GCL-2022-1034-01, 13 July 2022).

## 3. Results

### 3.1. Baseline Characteristics of the Subjects

During the study period, 9222 Korean adults (4577 men and 4645 women) with a median age of 62.8 years (interquartile range, IQR 54.5 to 71.8 years) underwent lipid profile tests using the Lipoprint LDL system. Baseline characteristics of the study subjects are summarized in Table 1. Among age groups, adults 60 to 69 years were the most prevalent in this study.

### 3.2. Lipid Test Results

According to NCEP ATP III criteria, the prevalence of hypercholesterolemia (total cholesterol ≥ 240 mg/dL) was 7.8%, low HDL (major risk of coronary heart disease) < 40 mg/dL was 12.9%, and high LDL ≥ 160 mg/dL was 0.5%. The overall prevalence of the sdLDL predominant non-A phenotype of LDL was 46.8% of the study subjects.

The prevalence of hyperlipidemia (high total cholesterol, low HDL, and high LDL) according to the NCEP ATP III criteria was significantly different between men and women (all *p* < 0.001). The prevalence of the sdLDL predominant non-A phenotype of LDL was significantly higher in men than in women (*p* < 0.001).

**Figure 1:**
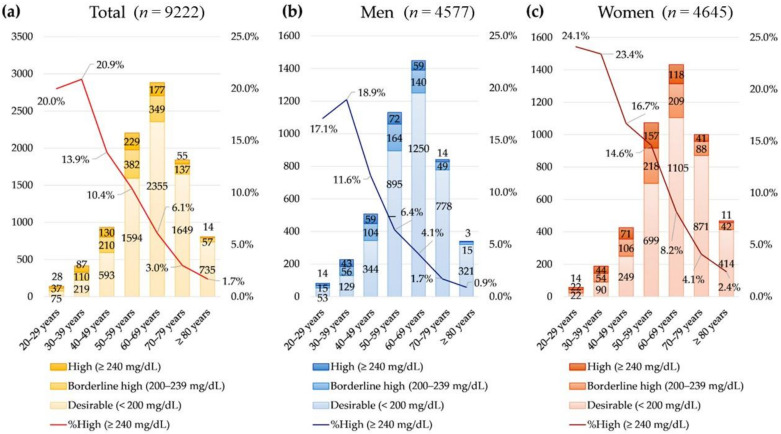
The prevalence of hypercholesterolemia (high total cholesterol ≥ 240 mg/dL) (**a**) in the total population, (**b**) in men, and (**c**) in women.

**Figure 2:**
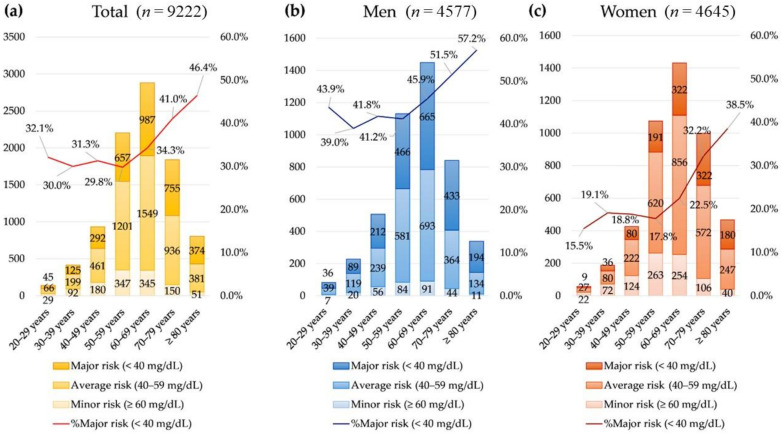
The prevalence of low HDL (HDL < 40 mg/dL) (**a**) in the total population, (**b**) in men, and (**c**) in women.

The prevalence of hyperlipidemia (high total cholesterol, low HDL, and high LDL) according to NCEP ATP III criteria was significantly different among age groups (all *p* < 0.001, Figure 1 and Figure 2). The prevalence of hypercholesterolemia (total cholesterol ≥ 240 mg/dL) was highest in patients younger than 40 years (20.7%) and decreased as age increased. Meanwhile, the prevalence of low LDL (major risk of coronary heart disease) was highest in patients older than 70 years (42.7%).

**Figure 3:**
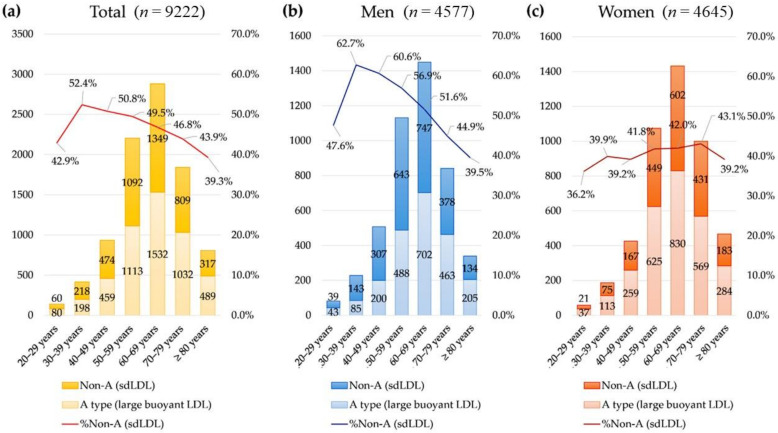
The prevalence of the small dense LDL predominant non-A phenotype (mean LDL particle size < 268 Å) (**a**) in the total population, (**b**) in men, and (**c**) in women.

The prevalence of the sdLDL dominant non-A phenotype was significantly different by sex and age group (all *p* < 0.001). In women, the prevalence of the sdLDL dominant non-A phenotype was stable across age groups. In men, the prevalence of the sdLDL dominant non-A phenotype was highest in men aged 30 to 39 years and decreased as age increased (Figure 3).

### 3.3. Presence of Small Dense LDL in Addition to Conventional Lipids

**Figure 4:**
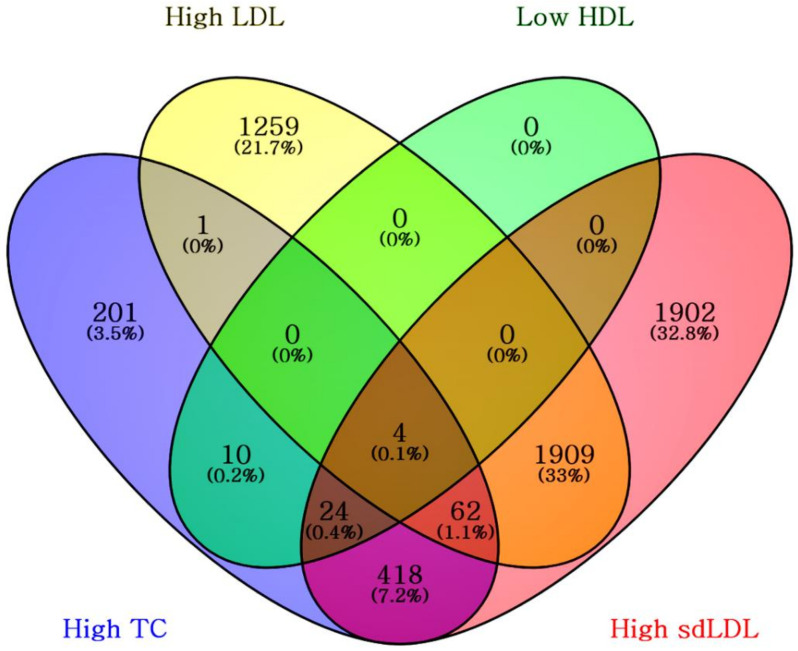
Venn diagram for the 5790 subjects having major risk factors for coronary heart disease; high total cholesterol (TC) ≥ 240 mg/dL, high LDL ≥ 160 mg/dL, low HDL < 40 mg/dL, and phenotype non-A predominant small dense LDL (sdLDL) with a mean LDL particle size < 268 Å.

Among the 9222 study subjects, 5790 had at least one high risk factor among the four lipid tests (total cholesterol, HDL, LDL, and sdLDL, Figure 4). Approximately 0.1% of the 5790 patients who exhibited high risk for at least one type of lipid test demonstrated high risk for all four lipid results (total cholesterol ≥ 240 mg/dL, HDL < 40 mg/dL, and LDL ≥ 160 mg/dL). Roughly 32.8% of the study subjects possessed lipid test results that did not exhibit an increased risk except for sdLDL (only the sdLDL predominant non-A phenotype as a risk factor).

## 4. Discussion

In this study, we investigated the prevalence of hyperlipidemia and sdLDL predominant patients in a large Korean population visiting local clinics and hospitals by sex and age group. The prevalence of hyperlipidemia and patients with the sdLDL predominant non-A phenotype significantly differed by sex and age group. These findings were comparable with previous studies in the Korean population [8,17,18].

According to the dyslipidemia factsheet in Korea 2020, the crude prevalence of hypercholesterolemia increased from 9.0% to 20.7% between 2007 and 2018 [18]. According to the fact sheet, the prevalence of hyper LDL cholesterolemia and hypo HDL cholesterolemia was 19.2% and 17.7%, respectively, in 2016 and 2018 [18]. In the present study, the prevalence of hypercholesterolemia, hyper LDL cholesterolemia, and hypo HDL cholesterolemia was 7.8%, 0.5%, and 12.9%, respectively, which were less than reported in the factsheet [18]. The reason for this discrepancy might be the population characteristics of the subjects. For example, LDL subfraction tests in this study were performed on patients with high risk of atherosclerotic diseases in this Korean population. In addition, approximately one-third of all patients exhibited the sdLDL predominant non-A phenotype with lipid profiles (total cholesterol, HDL, and LDL) not classified as high risk. Considering that roughly half of the study subjects had the sdLDL predominant non-A phenotype with normal LDL, utilization of sdLDL testing using LDL particle size analysis may provide further information for risk assessment of atherosclerotic cardiovascular diseases in addition to conventional lipid profile tests.

The numbers of patients tested for sdLDL in Korea were reviewed through a public database, the Healthcare Bigdata Hub by the HIRA, using the test code for D2640 (available at: http://opendata.hira.or.kr/op/opc/olapDiagBhvInfo.do, accessed on 18 July 2022). The utilization of sdLDL tests by LDL particle size analysis in Korea has significantly increased. According to the database, the number of patients undergoing these tests in Korea increased from approximately 8400 to 26,370 patients from 2018 to 2021. According to the database, the most prevalent population using this test was patients aged 60 to 69 years, comparable with the present study population. Although the public database of the Healthcare Bigdata Hub by HIRA estimated the number of patients with measured sdLDL in Korea, the prevalence of increased sdLDL level was not available. Considering that the number of tests performed in Green Cross Laboratories was approximately one-third of all reimbursed tests in Korea in 2021, the results of the present study may be representative of the utilization of sdLDL tests in Korea.

A limitation of this study is the lack of detailed clinical information about hypercholesterolemia and atherosclerotic cardiovascular disease, such as comorbidities (diabetes, obesities, and hypertension, etc.), social behaviors (smoking and alcohol consumption, etc.), and medications. Considering that the prevalence of hyper LDL cholesterolemia has been reported to be approximately 25.7% and 43.7% in Korean men and women aged 60 years or older, respectively, with those patients potentially taking lipid-lowering drug treatments, the results of this study should be interpreted with caution [18]. The exclusion of duplicate measures may also be a limitation of this study. However, the large number of subjects analyzed in this study is a strength. The results of this study could be helpful in allowing physicians to predict the overall prevalence of sdLDL predominant patients at high risk of atherosclerotic cardiovascular diseases in the adult Korean population. Future studies with detailed clinical histories of dyslipidemia and factors associated with atherosclerotic diseases are necessary.

## 5. Conclusions

In conclusion, we investigated the prevalence of hyperlipidemia and sdLDL predominant patients in a large Korean population by visiting local clinics and hospitals. Considering that approximately one-third of patients had the sdLDL predominant non-A phenotype without any other risk factor of lipid profiles, the use of sdLDL testing by LDL particle size analysis may provide additional information in identifying patients at high risk of atherosclerotic cardiovascular diseases. Future studies which clarify the clinical significance of this test using a large number of subjects from the Korean population are needed.

## Figures and Tables

**Table 1:** Baseline characteristics of the subjects.

Characteristics		Total (*n* = 9222)	Men (*n* = 4577)	Women (*n* = 4645)
Age, years (median, IQR)		62.8 (54.5 to 71.8)	62.0 (53.2 to 70.5)	63.7 (55.8 to 73.0)
Age group (*n*, %)	20–29 years	140 (1.5%)	82 (1.8%)	58 (1.2%)
	30–39 years	416 (4.5%)	228 (5.0%)	188 (4.0%)
	40–49 years	933 (10.1%)	507 (11.1%)	426 (9.2%)
	50–59 years	2205 (23.9%)	1131 (24.7%)	1074 (23.1%)
	60–69 years	2881 (31.2%)	1449 (31.7%)	1432 (30.8%)
	70–79 years	1841 (20.0%)	841 (18.4%)	1000 (21.5%)
	≥80 years	806 (8.7%)	339 (7.4%)	467 (10.1%)
Lipid test (median, IQR)	Total cholesterol, mg/dL	160 (136 to 193)	153 (131 to 185)	167 (142 to 201)
	HDL cholesterol, mg/dL	44 (37 to 52)	41 (35 to 48)	47 (40 to 56)
	LDL cholesterol, mg/dL	51 (38 to 70)	49 (37 to 67)	53 (40 to 74)
	Small dense LDL cholesterol, mg/dL	4 (2 to 11)	5 (2 to 12)	4 (1 to 10)
	Mean LDL absorbance, Å	268 (264 to 270)	267 (263 to 270)	269 (265 to 271)
**NCEP ATP III classification (*n*, %)**				
Total cholesterol	Desirable (<200 mg/dL)	7220 (78.3%)	3770 (82.4%)	3450 (74.3%)
	Borderline high (200–239 mg/dL)	1282 (13.9%)	543 (11.9%)	739 (15.9%
	High (≥240 mg/dL)	720 (7.8%)	264 (5.8%)	456 (9.8%)
HDL cholesterol	Low (major risk, <40 mg/dL)	1194 (12.9%)	313 (6.8%)	881 (19.0%)
	Average risk (40–59 mg/dL)	4793 (52.0%)	2169 (47.4%)	1140 (24.5%)
	High (minor risk, ≥60 mg/dL)	3235 (35.1%)	2095 (45.8%)	4209 (90.6%)
LDL cholesterol	Optimal (<100 mg/dL)	8517 (92.4%)	4308 (94.1%)	4209 (90.6%)
	Near optimal/above optimal (100–129 mg/dL)	552 (6.0%)	222 (4.9%)	330 (7.1%)
	Borderline high (135–159 mg/dL)	115 (1.2%)	36 (0.8%)	79 (1.7%)
	High (160–189 mg/dL)	24 (0.3%)	9 (0.2%)	15 (0.3%)
	Very high (≥190 mg/dL)	14 (0.2%)	2 (<0.1%)	12 (0.3%)
LDL phenotype (*n*, %)	Large buoyant LDL predominant (A type, ≥268 Å)	4903 (53.2%)	2186 (47.8%)	2717 (58.5%)
	Small dense LDL predominant (non-A type, <268 Å)	4319 (46.8%)	2391 (52.2%)	1928 (41.5%)

Abbreviation: IQR, interquartile range.

## Data Availability

The datasets generated and analyzed during the current study are available from the corresponding authors on reasonable request.
